# Stationäre Versorgungskapazitäten in der Kinder- und Jugendpsychiatrie – Zunahme der Akutaufnahmen während der COVID-19 Pandemie?

**DOI:** 10.1007/s40211-022-00423-2

**Published:** 2022-07-11

**Authors:** Kathrin Sevecke, Anna Wenter, Maximilian Schickl, Mariella Kranz, Nikola Krstic, Martin Fuchs

**Affiliations:** 1 Department für Kinder- und Jugendpsychiatrie, Medizinische Universität Innsbruck, Christoph-Probst-Platz 1, 6020 Innsbruck, Österreich; 2 Institut für Psychologie, Leopold-Franzens-Universität Innsbruck, Bruno-Sander-Haus, Innrain 52f, 6020 Innsbruck, Österreich; 3 Institut für Psychologie, UMIT – Private Universität für Gesundheitswissenschaften, Medizinische Informatik und Technik, Eduard-Wallnöfer-Zentrum 1, 6060 Hall in Tirol, Österreich

**Keywords:** COVID-19, Psychische Gesundheit, Kinder- und Jugendpsychiatrie, Akutaufnahmen, Suizidalität, COVID-19, Mental health, Child and adolescent psychiatry, Emergency admissions, Suicidality

## Abstract

Die COVID-19 Pandemie und die damit einhergehenden Eindämmungsmaßnahmen haben sich auf die psychische Gesundheit der Kinder und Jugendlichen ausgewirkt. In der vorliegenden Studie wurden die Akutaufnahmen der ersten beiden Corona-Jahre (2020/21) an der Abteilung für Kinder- und Jugendpsychiatrie Hall i. T./Innsbruck retrospektiv analysiert und mit den zwei Jahren vor der COVID-19 Pandemie (2018/19) verglichen. 2020 gab es keine Veränderung in der Gesamtzahl der Akutaufnahmen im Vergleich zum Vor-Corona-Jahr 2019, im Jahr 2021 stiegen die Akutaufnahmen hingegen um 40,1 %. Das Geschlechterverhältnis von 65,4 % Mädchen zu 34,6 % Jungen in den Vor-Corona-Jahren blieb im Jahr 2020 unverändert. 2021 stieg der Mädchenanteil auf 74,4 %. In der COVID-19 Pandemie nahm die akute Suizidalität zu (+48,3 %), wohingegen die Fremdaggression abnahm (−51,0 %). Akute Intoxikationen haben im ersten Corona-Jahr zugenommen und dann 2021 wieder abgenommen. Die vorliegenden Studienergebnisse zeigen, dass der Mental-Health-Bedarf bei Kindern und Jugendlichen im Verlauf der COVID-19 Pandemie anstieg und sich dies auch in der Kinder- und Jugendpsychiatrie deutlich bemerkbar machte. Den gestiegenen Anforderungen muss nun mit entsprechenden Versorgungs- und Präventionsmaßnahmen sowie ausreichenden kinder- und jugendpsychiatrischen Bettenkapazitäten begegnet werden, um längerfristige psychosoziale Auswirkungen der COVID-19 Pandemie bestmöglich abzufedern.

## Abstract (en)

The COVID-19 pandemic and the accompanying containment measures have had a fundamental impact on the mental health of children and adolescents. In the present study, the emergency admissions of the first two Corona years (2020/21) at the Department for Child and Adolescent Psychiatry Hall i. T./Innsbruck were retrospectively analysed and compared with the two years before the COVID-19 pandemic (2018/19). There was no change in the total number of emergency admissions in 2020 compared to 2019 before Corona, but in 2021 emergency admissions increased by 40.1%. The pre-Corona (2018/19) gender ratio of 65.4% girls to 34.6% boys did not change in 2020. In 2021, the proportion of girls increased to 74.4%. In the COVID-19 pandemic, acute suicidality increased (+48.3%) while aggression decreased (−51.0%). Acute intoxications increased in the first Corona year and decreased again in 2021. The present study results show that the mental health needs of children and adolescents increased significantly in the course of the COVID-19 pandemic and that this was also reflected in child and adolescent psychiatry. The increased demands must now be met with appropriate care and prevention measures as well as sufficient child and adolescent psychiatric bed capacities in order to mitigate the longer-term psychosocial effects of the COVID-19 pandemic as best as possible.

## Hintergrund

Vor mehr als zwei Jahren wurde der Ausbruch der Coronavirus-Erkrankung (COVID-19) zur Pandemie erklärt [42]. In den Jahren 2020 und 2021 reagierten Regierungen weltweit mit spezifischen Eindämmungsmaßnahmen, so auch die österreichische Regierung. Am 16. März 2020 trat der erste bundesweite Lockdown mit Ausgangsbeschränkungen, Schließung von Geschäften abseits der Grundversorgung und Einstellung des Präsenzunterrichts in Kraft [35]. Auch an den Kliniken wurden bisher beispiellose Maßnahmen ergriffen: Triage-Zelte wurden aufgebaut, nicht dringende Operationen wurden verschoben und Besuche an den Krankenanstalten waren nicht gestattet [26]. An der Kinder- und Jugendpsychiatrie (KJP) Hall i. T./Innsbruck wurden Gruppen- und Außenaktivitäten sowie Besuche ausgesetzt bzw. waren diese nur in Ausnahmefällen möglich. Die Tagesklinik wurde zeitweise geschlossen, während die ambulante und stationäre Pflichtversorgung zu jedem Zeitpunkt sichergestellt waren. Ab Mitte April 2020 wurden in Österreich zahlreiche Maßnahmen allmählich wieder aufgehoben [35], allerdings kam es nach dem relativ ruhigen Sommer 2020 im November 2020 zu einem zweiten Lockdown [33]. Sinkende Infektionszahlen ermöglichten im Frühling und Sommer 2021 wieder Lockerungen, bis im Herbst 2021 eine vierte Infektionswelle Österreich erfasste, die am 22. November 2021 in einen erneuten Lockdown mündete [34].

Internationale Metaanalysen zeigen negative Auswirkungen der COVID-19 Pandemie und der damit einhergehenden Eindämmungsmaßnahmen auf die psychische Gesundheit von Kindern und Jugendlichen, beispielsweise gestiegene Raten bei Angst- und depressiven Symptomen [36]. Die deutsche COPSY-Studie (COrona und PSYche) [37] liefert einen repräsentativen, längsschnittlichen Pre-Post-Vergleich: Die Anzahl der Kinder und Jugendlichen mit einer niedrigen Lebensqualität stieg im Frühling 2020 und Winter 2020/21 deutlich an und verbesserte sich im Herbst 2021 nur leicht. Auch Angstzustände und depressive Symptome nahmen im Frühling 2020 und Winter 2020/21 zu, gefolgt von einer nur leichten Verbesserung im Herbst 2021. Kinder aus bildungsfernen Familien, mit engen Wohnverhältnissen, Migrationshintergrund und elterlichen psychischen Problemen waren in dieser Studie besonders von negativen Auswirkungen betroffen [37]. Als ein weiterer Risikofaktor gilt das weibliche Geschlecht [14]. Pieh et al. [28] befragten im Februar 2021 online 3052 österreichische Jugendliche ab 14 Jahren: 55 % wiesen klinisch relevante depressive Symptome auf, 47 % Angstsymptome, 23 % Schlaflosigkeit, 60 % Essstörungssymptome und 37 % Suizidgedanken.

Es ist davon auszugehen, dass die sekundären Konsequenzen der Eindämmungsmaßnahmen (z. B. soziale Isolation, eingeschränkte Unterstützungsangebote) das Risiko für psychische Notfallsituationen erhöht haben und sich somit auf die stationäre Inanspruchnahme an Kinder- und Jugendpsychiatrien ausgewirkt haben. Das britische *Mental Health Network* berichtete im August 2021, dass aufgrund der Zunahme an Vorstellungen großer Druck und Bettenengpässe an Kinder- und Jugendpsychiatrien bestehen [22]. Im Oktober 2021 wurde in den Vereinigten Staaten ein nationaler Notstand im Bereich der seelischen Kinder- und Jugendgesundheit ausgerufen, nachdem ein dramatischer Anstieg an Notfallvorstellungen beobachtet worden war [2].

Erste Studien beschäftigen sich mit der Inanspruchnahme der Kinder- und Jugendpsychiatrien während der COVID-19 Pandemie. Eine multizentrische Studie mit Daten aus zehn Ländern (u. a. Österreich, England) zeigte eine sinkende Anzahl von kinder- und jugendpsychiatrischen Akutvorstellungen zu Beginn der Pandemie im März/April 2020 im Vergleich zum März/April des Vor-Corona-Jahres 2019. Der Schwerpunkt dieser Studie lag auf dem Thema Selbstverletzungen: Der Anteil der Patien_innen, die Selbstverletzungen aufwiesen, stieg von 50 % im Jahr 2019 auf 57 % im Jahr 2020 [27]. In einer italienischen Studie [7] wurden die ersten acht Wochen des ersten Lockdowns mit derselben Zeitperiode im Jahr 2019 verglichen: Es zeigte sich eine Reduktion der kinder- und jugendpsychiatrischen Notfälle von 46,2 %. Auch in US-amerikanischen Studien zeigte sich zunächst eine Reduktion der kinder- und jugendpsychiatrischen Akutvorstellungen in den ersten Monaten der COVID-19 Pandemie [19, 39]. Spätere Studienergebnisse weisen darauf hin, dass die kinder- und jugendpsychiatrischen Akutvorstellungen nach einem anfänglichen, Lockdown-bedingten Rückgang in der zweiten Jahreshälfte 2020 anstiegen [6, 18].

Während Ougrin et al. [27] den Fokus auf die Untersuchung von selbstverletzendem Verhalten legten, haben andere Studien die kinder- und jugendpsychiatrischen Akutvorstellungen in Hinblick auf Suizidalität untersucht. Hill et al. [15] fanden höhere Raten von Suizidgedanken im März und Juli 2020 und mehr Suizidversuche im Februar, März, April und Juli 2020 im Vergleich zu denselben Monaten im Jahr 2019. Yard et al. [44] berichteten, dass im Frühling 2020 weniger Akutvorstellungen wegen Suizidversuchen im Vergleich zum Frühling 2019 verzeichnet wurden. Im Sommer 2020 nahmen die Akutvorstellungen wegen Suizidversuchen zu und blieben über den restlichen Studienzeitraum bis Mai 2021 erhöht. Im Winter 2021 waren die Akutvorstellungen wegen Suizidversuchen bei den Mädchen um 50,6 % höher als im Winter 2019, bei den Jungen stiegen die Akutvorstellungen hingegen lediglich um 3,7 % [43]. Der höhere Mädchenanteil zeigte sich nicht nur bei den Akutvorstellungen aufgrund von Suizidversuchen: Krass et al. [17] berichteten, dass der Anteil der weiblichen Patient_innen bei den kinder- und jugendpsychiatrischen Akutvorstellungen in der COVID-19 Pandemie (62,6 % Mädchen) allgemein höher war als im Zeitraum Januar 2018 bis März 2020 (56,8 % Mädchen).

Studien berichten Ergebnisse aus der frühen Corona-Pandemie-Zeit (erstes Corona-Jahr 2020), wohingegen mittelfristige Auswirkungen auf Zahl und Art von psychiatrischen Notfällen bei Kindern und Jugendlichen noch nicht bekannt sind. Ein besseres Verständnis dieser mittelfristigen Auswirkungen ermöglicht eine Justierung der laufenden und zukünftigen Behandlungsangebote. Ziel der vorliegenden Studie ist es daher, die Akutaufnahmen an der KJP Hall i. T./Innsbruck der ersten beiden Corona-Jahre (2020/21) mit den Akutaufnahmen vor der COVID-19 Pandemie (2018/19) zu vergleichen. Ausgehend von der bestehenden Studienlage nehmen wir an, dass sich in der COVID-19 Pandemie – nach einer anfänglichen Reduktion – ein Anstieg in den kinder- und jugendpsychiatrischen Akutaufnahmen zeigt. Zudem nehmen wir an, dass Mädchen stärker betroffen sind und sich symptomabhängig unterschiedliche Veränderungen zeigen.

## Methoden

### Studiendesign

Es wurde eine retrospektive Analyse der stationären Akutaufnahmen an der KJP Hall i. T./Innsbruck durchgeführt. Die Akutaufnahmen der beiden Jahre vor Corona (2018/19) wurden mit den Akutaufnahmen der Jahre 2020 und 2021 (während Corona) verglichen. Die Auswertung und Publikation der Daten wurde von der Ethikkommission der Medizinischen Universität Innsbruck bewilligt (EK Nr.: 1400/2021).

### Setting

Für die vorliegende Studie wurden die elektronischen Patient_innenakten der Kinder und Jugendlichen (bis 18 Jahre) herangezogen, die aufgrund von Notfällen, die psychische Gesundheit betreffend, 2018 bis 2021 an der KJP Hall i. T./Innsbruck aufgenommen wurden. Es wurden sowohl Krisenaufnahmen (Patient_innen, die notfallmäßig vorstellig werden und akut behandlungsbedürftig sind) als auch Einweisungen nach § 8 oder § 9 des österreichischen Unterbringungsgesetzes (UbG) inkludiert [5]. Die KJP Hall i. T./Innsbruck hat akute Versorgungspflicht für das gesamte Bundesland Tirol (757.634 Einwohner, davon 147.692 Kinder und Jugendliche, Bettenmessziffer 2019: 0,06) und hat als einzige stationäre Einrichtung im Bundesland die Möglichkeit zur Unterbringung von Kindern und Jugendlichen nach dem UbG [12].

### Stichprobe

**Figure 1:**
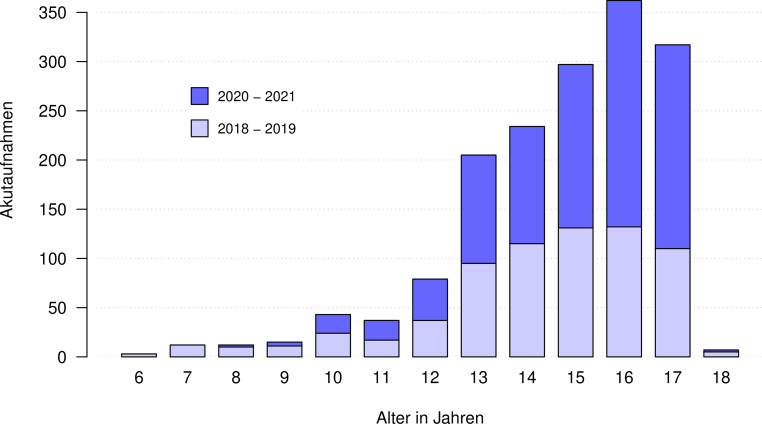

In den Jahren 2018 bis 2021 gab es an der KJP Hall i. T./Innsbruck insgesamt 1623 stationäre Akutaufnahmen (69,1 % Mädchen). Die Kinder und Jugendlichen waren im Alter von 6 bis 18 Jahren (*M* = 15,2, *SD* = 2,1). Abb. 1 zeigt die Altersstruktur der Patient_innen in den Vor-Corona-Jahren (2018/19) und in den Corona-Jahren (2020/21). Eine Behandlung im geschlossenen Bereich gemäß UbG fand bei 29,8 % der Patient_innen statt.

### Aufnahmegründe

Alle Notfallaufnahmen wurden, ausgehend von der bei der Aufnahme verfassten Anamnese, folgenden Aufnahmegründen zugeordnet (Mehrfachnennungen möglich): Suizidalität (Akute Suizidalität, Suizidgedanken distanziert), Nichtsuizidales selbstverletzendes Verhalten (NSSV), Akute Intoxikation, Fremdaggression, Restkategorie. Die Zuordnung zu den Kategorien erfolgte im Konsens zwischen den Autor_innen.

#### Suizidalität:

Suizidalität definiert sich über die Intention zu sterben und umfasst Suizidgedanken, Suizidankündigungen, Suizidpläne und Suizidversuche [8]. In der vorliegenden Studie wurde zwischen *Akuter Suizidalität *und *Suizidgedanken distanziert* unterschieden:

- *Akute Suizidalität: *Konkrete Suizidabsicht oder drängende Suizidgedanken mit unmittelbar drohender Suizidhandlung und zur Verfügung stehenden Mitteln [8].
- *Suizidgedanken distanziert: *Gedanken an den Tod, jedoch kann sich der/die Patient_in von akuter Suizidalität glaubhaft distanzieren (Kooperations- und Paktfähigkeit) [30].

#### Nichtsuizidales selbstverletzendes Verhalten (NSSV):

Direkte, repetitive, sozial nicht akzeptierte Schädigung von Körpergewebe [25] zum Zweck der Entlastung von negativen Gefühlen oder einem kognitiven Zustand bzw. um zwischenmenschliche Probleme zu lösen [11]. Dazu zählen z. B. Ritzen oder das Verbrennen der Körperoberfläche [31].

#### Akute Intoxikation:

Deutlicher Nachweis von kürzlich erfolgtem Substanzkonsum (z. B. Alkohol, Opioide) bzw. Intoxikationen mit Schmerzmitteln in (para)suizidaler Absicht. Die damit einhergehenden Symptome sind von ausreichendem Schweregrad, um Störungen von klinischer Relevanz zu verursachen [41].

#### Fremdaggression:

Unkontrollierte Aggression und daraus resultierende Fremdgefährdung. D. h., Patient_in zerstört Gegenstände oder verhält sich klar bedrohlich gegenüber anderen [9].

#### Restkategorie:

Aufnahmegründe, die inhaltlich nicht in die fünf zuvor definierten Kategorien fallen, z. B. dissoziativer Anfall, Psychose, Zwang.

### Statistik

Die Analysen wurden in R (V4.1.3) durchgeführt. Als Effektgröße wurde der Phi-Koeffizient (Φ) berechnet. Φ ≥ 0,1 bezeichnet kleine Effekte. Alle *p*-Werte wurden durch einen exakten Test nach Fisher-Freeman-Halton bestimmt. Eine Bonferroni-Signifikanzniveaukorrektur (*α* = 0,35 %) begrenzt den Fehler 1. Art für alle Vergleiche zusammen auf 5 %.

## Ergebnisse

### Gesamtzahl der Akutaufnahmen (2018–2021)

**Figure 2:**
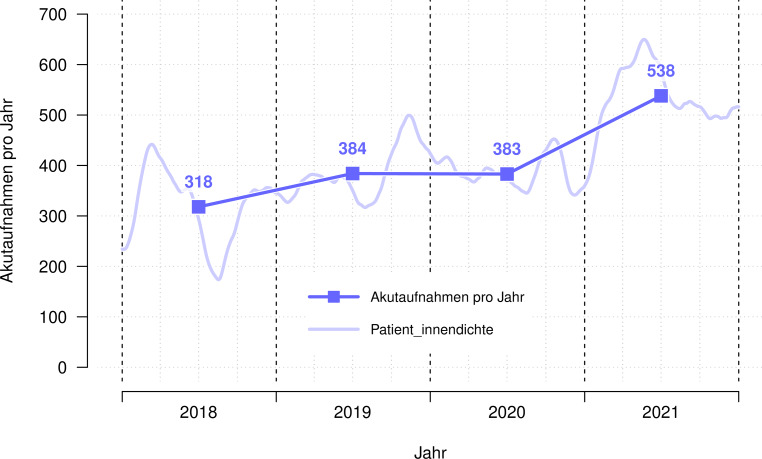

In den Jahren 2018 und 2019 gab es 318 und 384 Akutaufnahmen. Die 383 Akutaufnahmen im Jahr 2020 stellen keine Veränderung zum Vor-Corona-Jahr 2019 dar (Φ = 0,00, *p* = 0,971). Im Jahr 2021 sind die Akutaufnahmen im Vergleich zum Vor-Corona-Jahr 2019 hingegen um 40,1 % auf 538 angestiegen (Φ = 0,17, *p* < 0,001). Abb. 2 zeigt den zeitlichen Verlauf der Akutaufnahmen.

#### *Anmerkung*

Patient_innendichte = zu erwartende Anzahl der Akutaufnahmen pro Jahr, wenn die Häufigkeit das ganze Jahr so wäre wie zu diesem bestimmten Zeitpunkt.

### Geschlechterverhältnis

**Figure 3:**
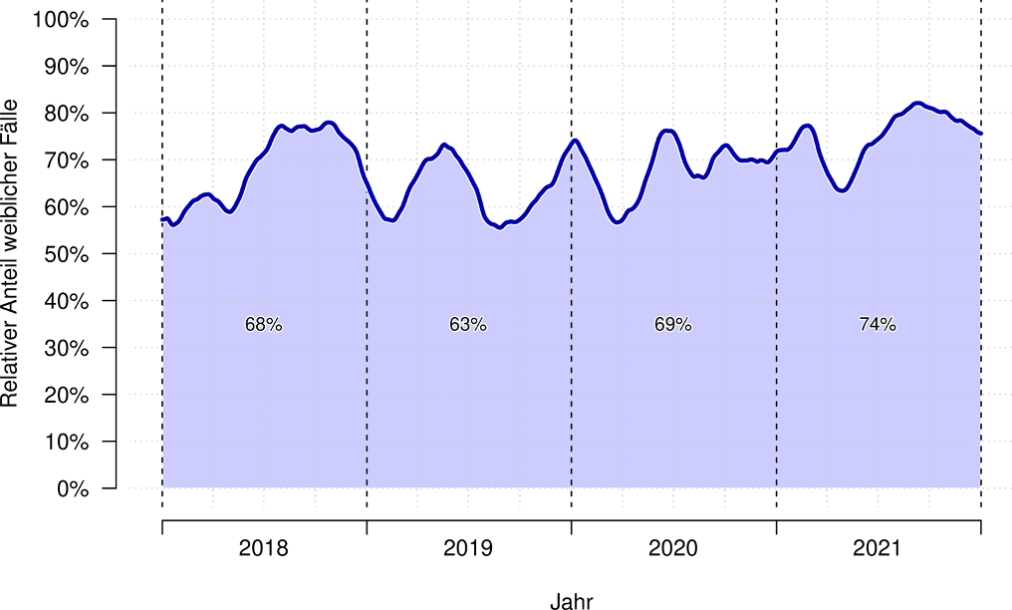

Das Geschlechterverhältnis von 65,4 % Mädchen zu 34,6 % Jungen in den Vor-Corona-Jahren 2018/19 blieb im Jahr 2020 unverändert (68,7 % Mädchen, Φ = −0,03, *p* = 0,274). Im Jahr 2021 ist der Mädchenanteil jedoch angestiegen (74,4 %, Φ = −0,10, *p* < 0,001). Drei von vier Akutaufnahmen im Jahr 2021 waren Mädchen. Abb. 3 zeigt das Geschlechterverhältnis über die Jahre.

### Aufnahmegründe im Detail

**Figure 4:**
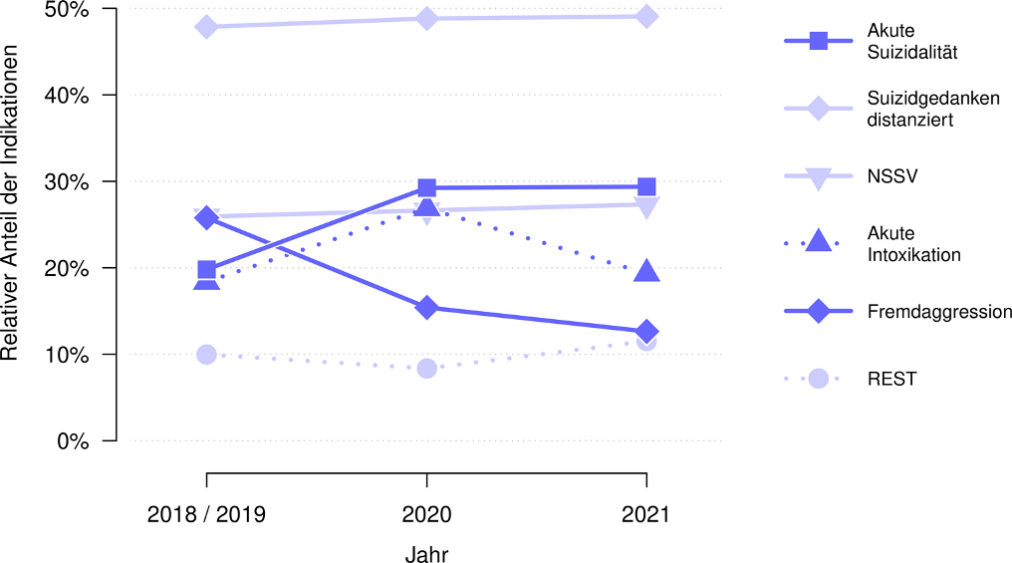

In den Corona-Jahren 2020/21 nahm die akute Suizidalität zu, die Fremdaggression ab. Akute Intoxikationen nahmen im ersten Corona-Jahr zu und dann 2021 wieder ab. Der Anteil an suizidalen Gedanken, NSSV und anderen Gründen blieb unverändert. Abb. 4 zeigt den relativen Anteil der einzelnen Indikationen bei der Aufnahme im zeitlichen Verlauf. In der Folge werden die Ergebnisse zu den Aufnahmegründen im Detail dargestellt.

Akute Suizidalität zeigte sich bei den Akutaufnahmen der Jahre 2020 (29,2 %, Φ = 0,11, *p* < 0,001) und 2021 (29,4 %, Φ = 0,11, *p* < 0,001) häufiger als bei den Akutaufnahmen der Vor-Corona-Jahre 2018/19 (19,8 %). Dies entspricht einem Anstieg von 48,3 % in den Corona-Jahren.

Suizidale Gedanken stellten in den Jahren 2018/19 mit 47,9 % die häufigste Indikation dar. Hier gab es weder 2020 (48,8 %, Φ = 0,01, *p* = 0,762) noch 2021 (49,1 %, Φ = 0,01, *p* = 0,674) eine Veränderung.

Auch der relative Anteil an NSSV blieb 2020 (26,6 %, Φ = 0,01, *p* = 0,801) und 2021 (27,3 %, Φ = 0,02, *p* = 0,582) im Vergleich zu den Jahren 2018/19 (25,9 %) unverändert.

Akute Intoxikationen sind im Vergleich zu den Jahren 2018/19 (18,4 %) im Jahr 2020 gestiegen (26,9 %, Φ = 0,10, *p* = 0,001). 2021 kehrte der Anteil der akuten Intoxikationen wieder auf das Ursprungsniveau vor Ausbruch der COVID-19 Pandemie zurück (19,3 %, Φ = 0,01, *p* = 0,670).

Fremdaggression trat im Vergleich zu den Jahren 2018/19 (25,8 %) sowohl im Jahr 2020 (15,4 %, Φ = −0,12, *p* < 0,001) als auch im Jahr 2021 (12,6 %, Φ = −0,16, *p* < 0,001) seltener auf. Dies entspricht einer Abnahme von −51,0 % in den Corona-Jahren.

Bei 10,0 % der Fälle wurde in den Jahren 2018/19 keine der fünf Indikationen angegeben, sondern andere Gründe (z. B. dissoziativer Anfall; siehe Methoden). Der Anteil dieser Restkategorie blieb in den Jahren 2020 (8,4 %, Φ = −0,03, *p* = 0,384) und 2021 (11,5 %, Φ = 0,02, *p* = 0,380) unverändert.

## Diskussion

Zusammenfassend zeigte sich in unserer Studie, dass es 2020 keine Veränderung in der Gesamtzahl der Akutaufnahmen im Vergleich zum Vor-Corona-Jahr 2019 gab. Im Jahr 2021 stiegen die Akutaufnahmen hingegen um 40,1 %. Das Geschlechterverhältnis von 65,4 % Mädchen zu 34,6 % Jungen in den Vor-Corona-Jahren 2018/19 blieb im Jahr 2020 unverändert. Im Jahr 2021 stieg der Mädchenanteil jedoch an. In der COVID-19 Pandemie nahm die akute Suizidalität zu, wohingegen die Fremdaggression abnahm. Akute Intoxikationen nahmen im ersten Corona-Jahr zu und dann 2021 wieder ab.

Es ist anzunehmen, dass die Angst vor einer möglichen Ansteckung mit dem damals neuartigen Virus zunächst dazu geführt hat, dass Familien zu Beginn der Pandemie die Klinik nach Möglichkeit mieden und die Anzahl der Akutaufnahmen im ersten Corona-Jahr somit nicht anstieg. Im März 2020 wurde in den österreichischen Medien kommuniziert, dass das Gesundheitswesen angesichts der Pandemie auf dringliche Fälle fokussieren müsse – zum einen um das System zu entlasten, zum anderen um Infektionen im direkten Kontakt zu vermeiden [13, 26]. Auch das *Pulling-Together* der Gesellschaft zu Beginn der Pandemie könnte dazu beigetragen haben, dass die kinder- und jugendpsychiatrischen Notfallsituationen zunächst nicht anstiegen. Der sogenannte *Pulling-Together-Effekt* besagt, dass Menschen, die gemeinsam eine herausfordernde Erfahrung durchleben, sich gegenseitig unterstützen, so die soziale Verbundenheit gestärkt wird und das *Pulling-Together* mit niedrigeren Suizidraten verbunden ist [16]. Während die Anzahl der Akutvorstellungen und -aufnahmen in anderen Studien [7, 39] zu Beginn der COVID-19 Pandemie sogar sank, blieb die Anzahl der Akutaufnahmen an der KJP Hall i. T./Innsbruck im Jahr 2020 gleich. Damit übereinstimmend, berichtete Mustafa [24], dass die psychiatrischen Notfallvorstellungen bei Erwachsenen im April 2020 im Vergleich zu vor der COVID-19 Pandemie gleichblieben, während die nicht-psychiatrischen Notaufnahmen in diesem Zeitraum abnahmen.

Im Jahr 2021 stieg dann die Anzahl der Akutaufnahmen an der KJP Hall i. T./Innsbruck, was mit den in Studien [4, 37, 43] berichteten negativen Auswirkungen der COVID-19 Pandemie auf die psychische Gesundheit von Kindern und Jugendlichen einhergeht. Ähnlich den vorliegenden Studienergebnissen berichteten Ambrosetti et al. [1] nach dem ersten Lockdown eine Zunahme der psychiatrischen Notfallvorstellungen bei Erwachsenen um 21 % und sprachen von einem *Rebound-Effekt* in der Post-Lockdown-Zeit. Der Anstieg der kinder- und jugendpsychiatrischen Notfallaufnahmen nach der Akutphase der COVID-19 Pandemie könnte zum einen auf die zeitweise Schließung bzw. eingeschränkte Verfügbarkeit von Gesundheits- und Sozialdiensten, zum anderen auf die Unterbrechung von Psychotherapien und anderen routinemäßigen therapeutischen Kontakten zurückzuführen sein. Dadurch erhöhte sich das Risiko für eine klinische Verschlechterung des psychischen Gesundheitszustands. Zu nennen sind auch die Einschränkung der sozialen Kontakte und die Schulschließungen in der Pandemiezeit. In der repräsentativen deutschen COPSY-Studie (*CORona und PSYche*) [37] gaben in den ersten beiden Erhebungen (Mai/Juni 2020 und Dezember 2020/Jänner 2021) zirka 80 % der Kinder und Jugendlichen an, weniger soziale Kontakte als vor der Pandemie gehabt zu haben und etwa zwei Fünftel gaben Beeinträchtigungen in den Beziehungen zu ihren Freund_innen an. Mehr als die Hälfte der Teilnehmer_innen berichtete, dass der Schulbesuch und das Lernen schwieriger waren als vor der Pandemie [37]. Die im Jahr 2020 in Deutschland beobachtete Abnahme der Lebensqualität und die Zunahme von Verhaltensproblemen, Beeinträchtigungen in den Beziehungen zu Gleichaltrigen, Ängsten sowie depressiven und psychosomatischen Symptomen bei Kindern und Jugendlichen wurden im zweiten Pandemiejahr 2021 in Südtirol bestätigt [4]. In der Tiroler COVID-19 Kinderstudie [43] zeigten die Kindergarten- und Grundschulkinder im Dezember 2021 mehr internalisierende Probleme und posttraumatische Belastungssymptome als im März 2020. Darüber hinaus nahm laut der Metaanalyse von Piquero et al. [29] in der Krisenzeit die häusliche Gewalt zu. Loiseau et al. [20] berichteten von einem alarmierenden Anstieg der relativen Häufigkeiten von Krankenhausaufnahmen während des Lockdowns, die auf Kindesmisshandlung zurückzuführen waren. Es ist davon auszugehen, dass diese psychosozialen Belastungen zum Anstieg der Akutaufnahmen beigetragen haben.

Übereinstimmend mit der Studie von Krass et al. [17] stieg in unserer Studie im zweiten Jahr der Pandemie der Mädchenanteil bei den Akutaufnahmen. Es ist bekannt, dass das Hilfesuchverhalten und die Inanspruchnahme von Beratungen bei Männern/Jungen durchwegs niedriger sind als bei Frauen/Mädchen, insbesondere bei emotionalen Problemen. Empirische Daten zeigen, dass die niedrigeren Behandlungsraten bei Männern nicht durch einen besseren Gesundheitszustand zu erklären sind, sondern auf eine Diskrepanz zwischen Wahrnehmung des Bedarfs und dem Verhalten bei der Hilfesuche zurückzuführen sind [23]. Abgesehen davon zeigt sich in repräsentativen Studien zur psychischen Gesundheit von Kindern und Jugendlichen, dass geschlechtsspezifische Unterschiede bestehen, wobei Mädchen deutlich häufiger von psychischen Problemen betroffen sind als Jungen [40]. Bei einer retrospektiven Analyse der Akutvorsorgung an der KJP Salzburg in den Jahren 2016/17 waren 53,7 % der Patient_innen weiblich [10]. Auch zahlreiche COVID-19-Studien identifizierten das weibliche Geschlecht als einen Risikofaktor [14]. Die COPSY Studie und die Tiroler COVID-19 Kinderstudie zeigten, dass internalisierende Probleme, beispielsweise Angst und depressive Symyptome, in der Pandemie zunahmen [4, 37, 43] und diese betrafen häufiger Mädchen als Jungen [37].

In Übereinstimmung mit den Studienergebnissen aus dem Bundesland Salzburg (53,8 %) [10] stellten auch in der vorliegenden Studie suizidale Gedanken den häufigsten Vorstellungsgrund dar (2018/19: 47,9 %, 2020: 48,8 %, 2021: 49,1 %). In unserer Studie zeigten sich in der Corona-Pandemie Veränderungen in den relativen Anteilen der Aufnahmegründe. Akute Suizidalität stieg um 48,3 % im Vergleich zu den Vor-Corona-Jahren an. Der Rückgang in der Fremdaggression könnte als Gegenspieler betrachtet werden und u. a. auf die Eindämmungsmaßnahmen und die damit einhergehenden reduzierten sozialen Kontakte zurückzuführen sein. Korrespondierend mit unserer Studie berichteten Ambrosetti et al. [1] bei psychiatrischen Notfallvorstellungen von Erwachsenen nach dem Lockdown eine Zunahme von Suizidalität und psychomotorischer Unruhe, während Verhaltensstörungen weniger häufig auftraten. Auch bei Kindern und Jugendlichen wurde eine Zunahme der Akutvorstellungen und -aufnahmen aufgrund von Suizidgedanken bzw. -versuchen berichtet [15, 43]. Die soziale Isolation und Einschränkungen in der Behandlung psychischer Erkrankungen zu Beginn der Pandemie trugen zu einem *perfect storm for mental health* bei [38]. Neben Isolation und Einsamkeit sind eine Reihe anderer mit der Pandemie einhergehende Umstände (z. B. wirtschaftliche Einbußen, häusliche Gewalt, Stigmatisierung, Angst) hinlänglich als Risikofaktoren für die psychische Gesundheit (z. B. in Bezug auf Suizidalität) bekannt und trugen zu diesem *perfect storm* bei [3]. Während Chadi et al. [6] berichteten, dass die Akutvorstellungen wegen Substanzkonsum im Jahr 2020 unverändert im Vergleich zu den Vor-Corona-Jahren 2018/19 waren, wurden an der KJP Hall i. T./Innsbruck 2020 mehr akute Intoxikationen verzeichnet. 2021 kehrte der Anteil der akuten Intoxikationen wieder auf das Ursprungsniveau vor Ausbruch der COVID-19 Pandemie zurück. Lundahl und Cannoy [21] berichteten in einem Review, dass die Auswirkungen der COVID-19 Pandemie auf den jugendlichen Substanzkonsum noch nicht zur Gänze geklärt sind. Diesbezügliche Studien führten teilweise zu widersprüchlichen Ergebnissen. Ähnlich wie bei der Suizidalität ist allerdings auch hier davon auszugehen, dass in der Akutphase der COVID-19 Pandemie bereits bestehende Risikofaktoren für jugendlichen Substanzkonsum (z. B. eingeschränkte soziale und schulische Verbundenheit, Langeweile) verschärft wurden [21].

Die vorliegende Studie liefert einen wertvollen und detaillierten Einblick in die Notfallaufnahmen während der ersten zwei Jahre der COVID-19 Pandemie. Die KJP Hall i. T./Innsbruck ist eine der 12 Krankenhausabteilungen zur stationären kinder- und jugendpsychiatrischen Versorgung in Österreich und für die Versorgung des gesamten Bundeslandes Tirol zuständig [12]. Allerdings sollten unsere Ergebnisse unter Berücksichtigung einiger Limitationen betrachtet werden. Die Daten stammen aus einer einzigen Klinik und es wurden lediglich die stationären Akutaufnahmen in die Studie eingeschlossen. Des Weiteren wurde die Gesamtzahl der Akutaufnahmen in den Corona-Jahren 2020/21 mit dem Vor-Corona-Jahr 2019 verglichen. Auf eine Mittelung der Werte von 2018 und 2019 wurde verzichtet, weil die KJP im November 2017 von Innsbruck nach Hall i. T. umgezogen ist. 2018 – das erste Jahr am neuen Standort (einhergehend mit strukturellen Veränderungen) – stellte somit kein *normales Jahr *dar. Methodisch ist anzumerken, dass die Daten nicht systematisch durch standardisierte Bewertungsskalen oder Interviews erhoben wurden, sondern dass die Krankenakten retrospektiv nach zuvor definierten Aufnahmegründen kategorisiert wurden. Schließlich ist noch anzumerken, dass die Akutaufnahmen einmalige Ereignisse, nicht einzelne Personen darstellen und daher mehrere Akutaufnahmen ein- und derselben Person widerspiegeln können.

## Fazit für die Praxis

Mit unserer Studie konnten wir im Versorgungsraum Tirol einen mittelfristigen Anstieg der Inanspruchnahme von psychiatrischen Notfallbehandlungen bei Kindern und Jugendlichen zeigen. Der niedrige Bettenschlüssel führte in diesen Krisenzeiten zu Druck und Engpässen in der Versorgung akut behandlungsbedürftiger Kinder und Jugendlicher – wobei Akutaufnahmen jederzeit möglich waren, längerfristige stationäre Therapieplätze jedoch eine mehrmonatige Wartezeit bedeuteten. Den gestiegenen Anforderungen im Mental-Health-Bereich muss nun mit entsprechenden Versorgungs- und Präventionsmaßnahmen sowie ausreichenden kinder- und jugendpsychiatrischen Bettenkapazitäten sowie ambulanten Behandlungsmöglichkeiten begegnet werden. Das Monitoring der psychischen Gesundheit der Kinder und Jugendlichen in der Pandemie sowie die Förderung von Coping und Resilienz müssen längerfristig im Auge behalten werden. Insbesondere für eine flächendeckende und niederschwellige Suizidprävention sind ausreichend Mittel zur Verfügung zu stellen. Ausweitung und Verbesserung des Behandlungsangebotes zur Unterstützung der psychischen Gesundheit von Kindern und Jugendlichen (z. B. Erhöhung der Bettenmessziffer, Stellenausbau, vermehrte Verankerung niederschwelliger therapeutischer Angebote im Alltag von Kindern und Jugendlichen) (siehe auch [32]) sind notwendig, um die längerfristigen psychosozialen Auswirkungen der COVID-19 Pandemie möglichst gut abzufedern.
